# Hierarchical assembly of traditional chinese medicine-based hydrogels for wound healing

**DOI:** 10.1186/s12951-026-04504-0

**Published:** 2026-05-10

**Authors:** Yue-Yi Ren, Xin-Yi Yang, Yue-Ting Lin, Xu-Jia Hong, Jin-Sheng Zhang

**Affiliations:** 1 The Affiliated Traditional Chinese Medicine Hospital, Guangzhou Medical University, Guangzhou, 511436 China; 2 School of Pharmaceutical Sciences, Guangzhou Medical University, Guangzhou, 511436 China

**Keywords:** Traditional Chinese medicine-based hydrogels, Hierarchical assembly, Wound healing, Structure-activity relationship, Synergistic therapy

## Abstract

Chronic wound healing represents a significant clinical challenge, requiring multi-target intervention strategies due to the complex pathological microenvironment. Traditional Chinese Medicine (TCM) has a long wound care history and TCM-based hydrogels have evolved from single- to multi-component designs. However, existing reviews primarily focus on preparation methods, lacking a systematic analysis of how the hierarchical assembly of TCM components influences hydrogel structure, properties, and wound healing efficacy via structure–activity relationships. This review proposes a novel component-number hierarchy, defined as a systematic analytical framework classifying TCM-based hydrogels by active component count and assembly logic. This framework stratifies systems into four progressive hierarchical levels: single-component, dual-component, multi-component, and formula-mimetic systems. Specifically, formula-mimetic systems are defined as hydrogel platforms that mimic TCM’s “emperor-minister-assistant-courier” principle via rational hierarchical assembly of multi-components with well-defined roles, to replicate holistic multi-target synergistic effects in wound dressings. We summarize the driving forces for assembly, structural formation mechanisms, and key physicochemical properties across different hierarchical levels, and analyze how these properties govern functional performance at distinct stages of wound healing. By addressing current challenges and future directions, this review provides a theoretical basis and design principles for developing advanced intelligent TCM hydrogel dressings.

**Graphical Abstract:**

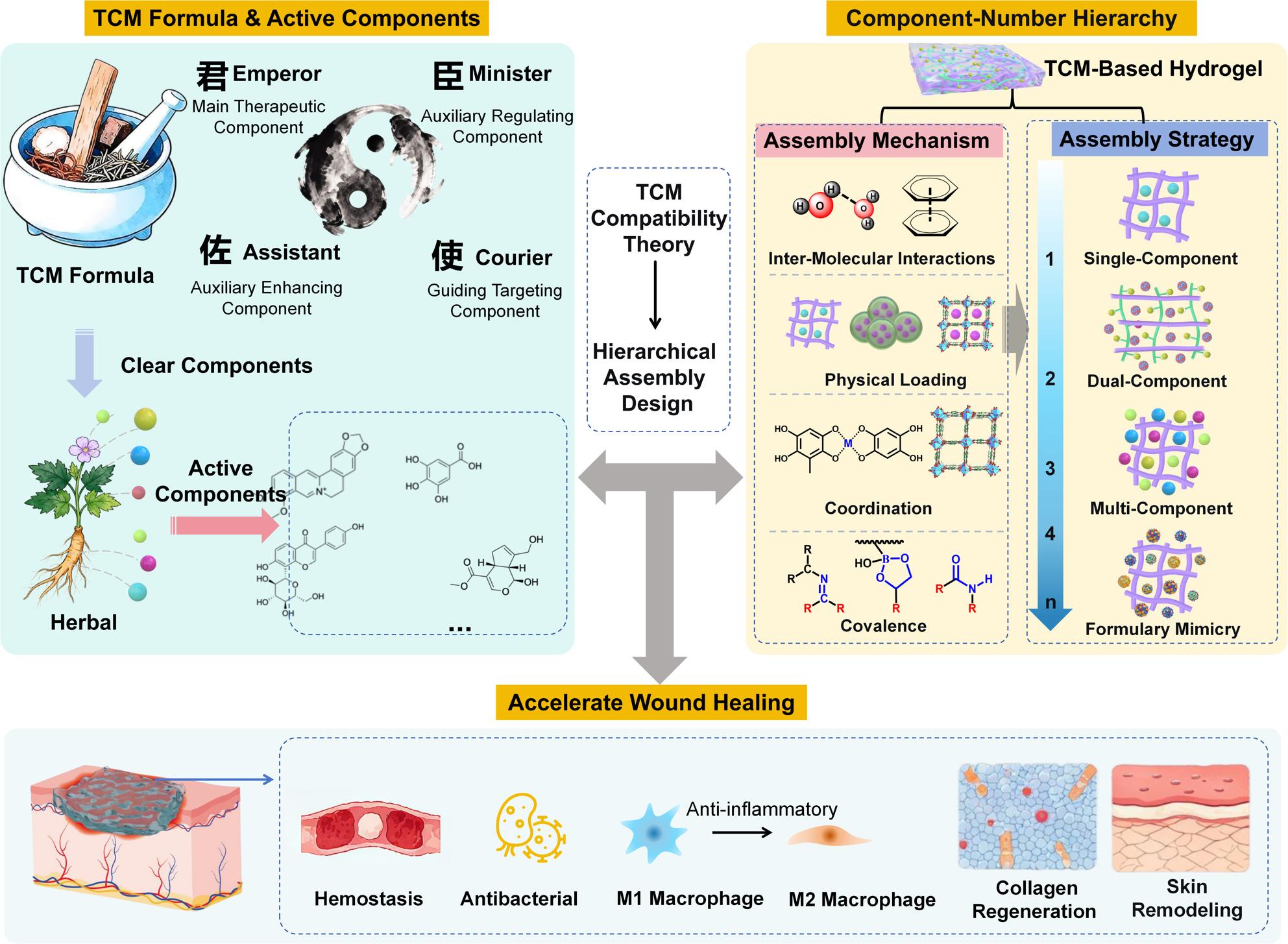

## Introduction

Chronic non-healing wounds, such as diabetic foot ulcers and pressure ulcers, impose a heavy burden on the global healthcare system due to their complex pathophysiological processes and increasing prevalence [1, 2]. These wounds not only severely impair patients’ quality of life but also are prone to secondary infections, significantly increasing the risk of amputation and even death [3–6]. Conventional dressings largely provide only physical protection and moisture retention, failing to address the multiple pathological hallmarks of chronic wounds, including persistent inflammation, recurrent infection, dysregulated extracellular matrix remodeling, and impaired regeneration [7–9]. Traditional Chinese medicine (TCM) exhibits unique potential in this field with its holistic perspective and systematic regulation [10, 11]. The “emperor-minister-assistant-courier” (EMAC) compatibility theory embodies a sophisticated approach to multi-component, multi-target therapy that aligns remarkably well with the complex, multi-stage process of wound healing [12]. Modern research has validated the therapeutic potential of numerous TCM-derived compounds, such as baicalin, berberine (Ber), astragalus polysaccharides, and gallic acid (GA). These compounds exert synergistic therapeutic effects by modulating key signaling pathways, including NF-κB, Nrf2, TGF-β, laying a robust pharmacological foundation for chronic wound intervention [13–15].

Most bioactive TCM compounds suffer from poor aqueous solubility, low physiological stability, rapid in vivo metabolism, and limited bioavailability. Meanwhile, traditional topical formulations are limited by uncontrolled drug release and poor patient compliance [16–19]. Hydrogels, which can mimic the extracellular matrix and provide a moist healing environment, have emerged as a highly designable drug delivery platform. It can enable the loading and control release of TCM components through physical encapsulation, coordination bonding, covalent grafting, and other methods [20–25].

In recent years, research on TCM hydrogels has shown a clear trend of evolving from single-component loading to multi-component synergistic systems. However, existing reviews have focused on summarizing preparation methods. They lack a systematic analysis of the principles, mechanisms, and structure-activity relationships that govern the assembly of TCM components at varying hierarchical levels and their resulting effects on wound healing. Understanding how to assemble multiple TCM components into one hydrogel is crucial. This achieves their traditional synergistic effects and promotes TCM modernization. It also helps develop a new generation of intelligent wound dressings.

**Fig. 1:**
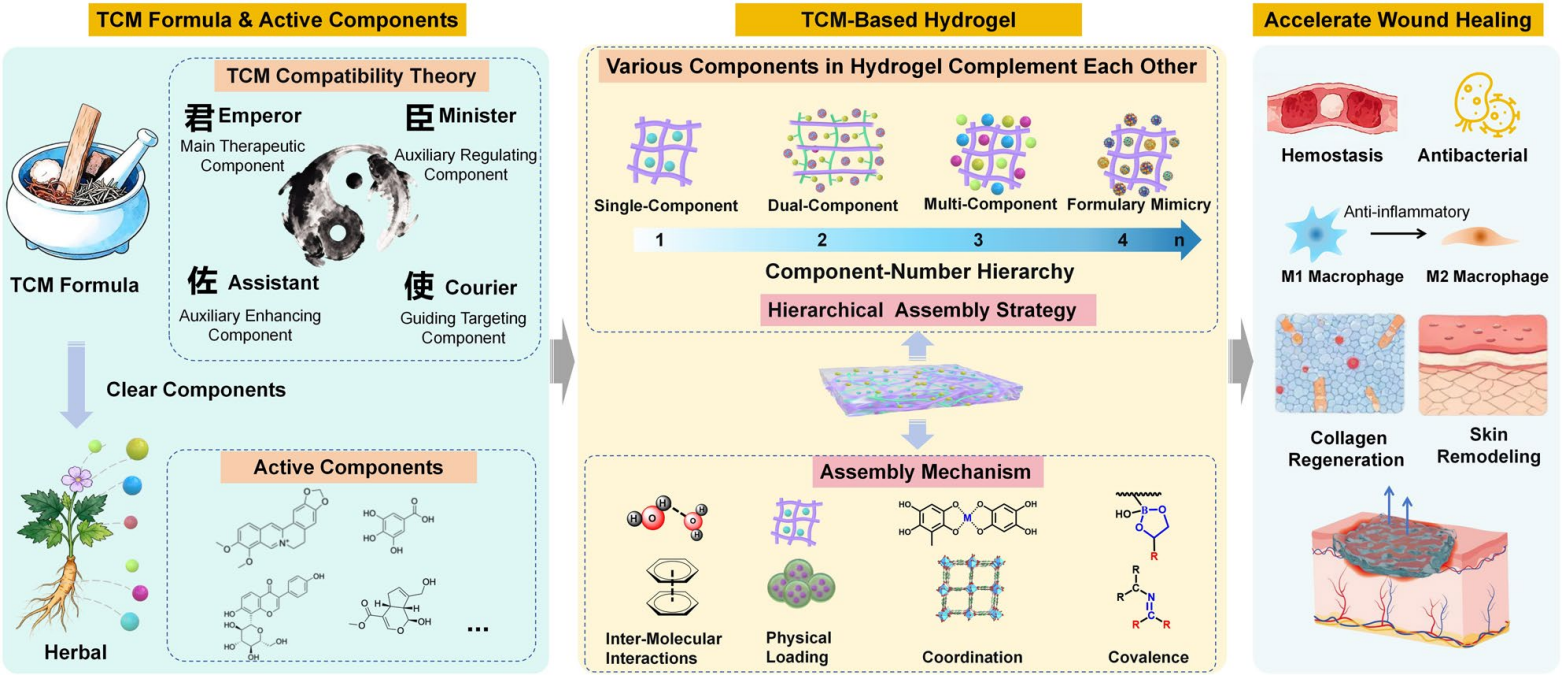
Schematic diagram of the hierarchical assembly strategy of TCM-based hydrogels and their application in wound healing

Therefore, this review proposes the “component-number hierarchy” as the core analytical framework (Fig. 1). Figure 1 systematically illustrates the translation of the classic EMAC compatibility theory into modern hydrogel material design logic, as well as the progressive four-level component-number hierarchy framework of this review. This framework establishes a complete logical chain between TCM synergistic principles, hierarchical assembly strategies, hydrogel network structures, and wound healing therapeutic efficacy. The assembly rules and mechanisms of TCM-based hydrogels assembled from different single-component, dual-component, multi-component, and formulary mimicry TCM are reviewed. The dominant assembly driving forces at different levels and the characteristics of the formed network structures are summarized. Further elaboration is provided on how these hydrogel systems target and intervene in each key stage of wound healing. Specifically, these systems promote early hemostasis, exert efficient anti-inflammatory effects during the inflammatory phase, accelerate collagen deposition and vascular regeneration in the proliferative phase, and optimize skin tissue remodeling in the remodeling phase [26–32]. This review aims to provide a theoretical foundation and practical roadmap for overcoming the limitations of traditional TCM compound preparations, and for the rational design of novel TCM-based hydrogel dressings with synergistic pharmacological effects, clear therapeutic mechanisms, and tailorable engineering properties.

## Single-component TCM hydrogels

**Fig. 2:**
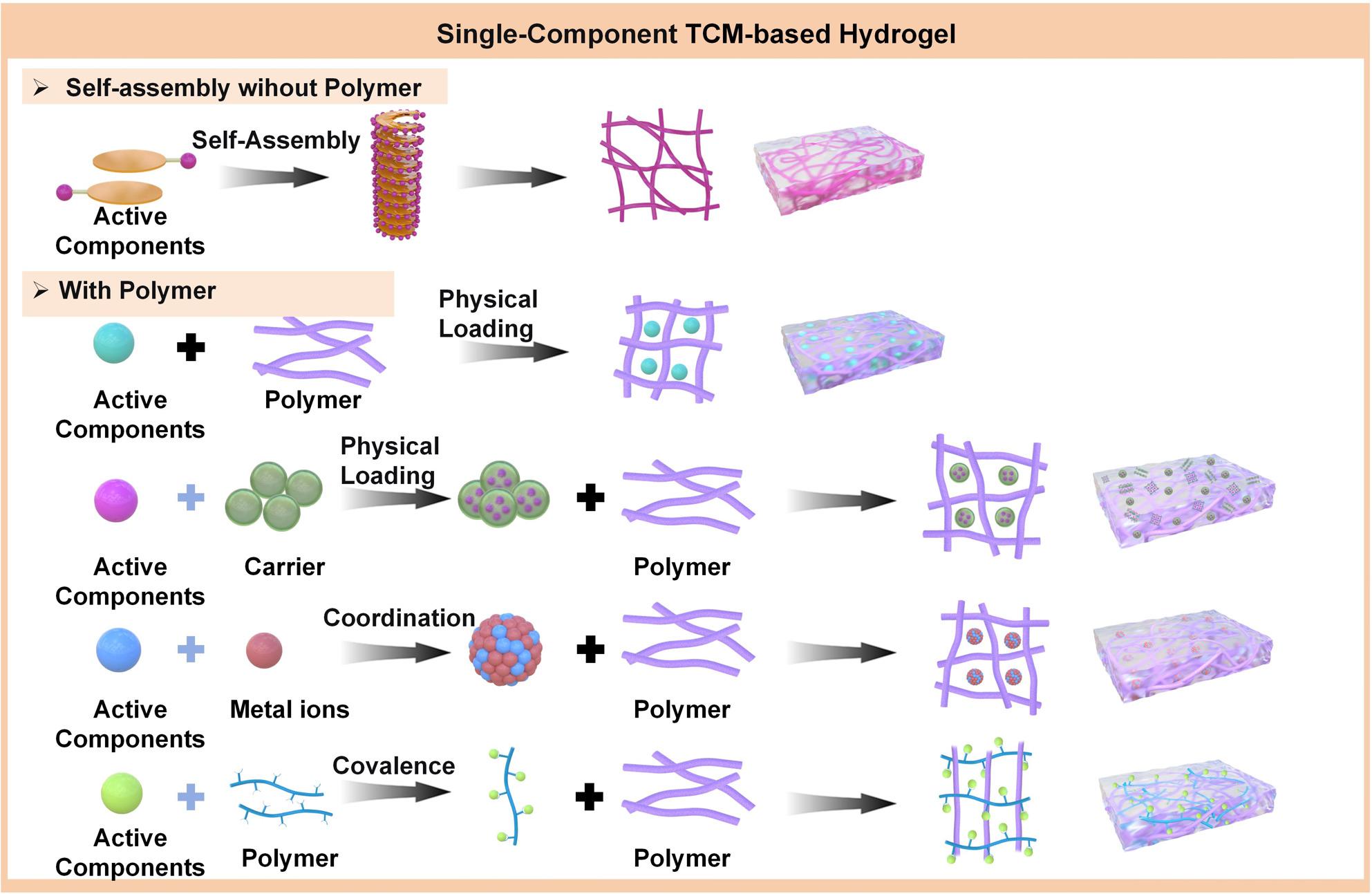
Classification of assembly strategies for single-component TCM-based hydrogels

As the first and most fundamental level of the component-number hierarchy framework (Fig. 1), single-component TCM-based hydrogel systems serve as the core research model for elucidating the independent assembly behavior, intrinsic structure-activity relationships, and therapeutic activity of individual TCM “Emperor” components. These systems thus lay the essential groundwork for the development of more complex synergistic systems. As shown in Fig. 2, these systems can be classified into two main categories based on their assembly mechanisms, self-assembly-dominated and polymer-integrated types.

### Self-assembly dominated single-component TCM-based hydrogels

**Fig. 3:**
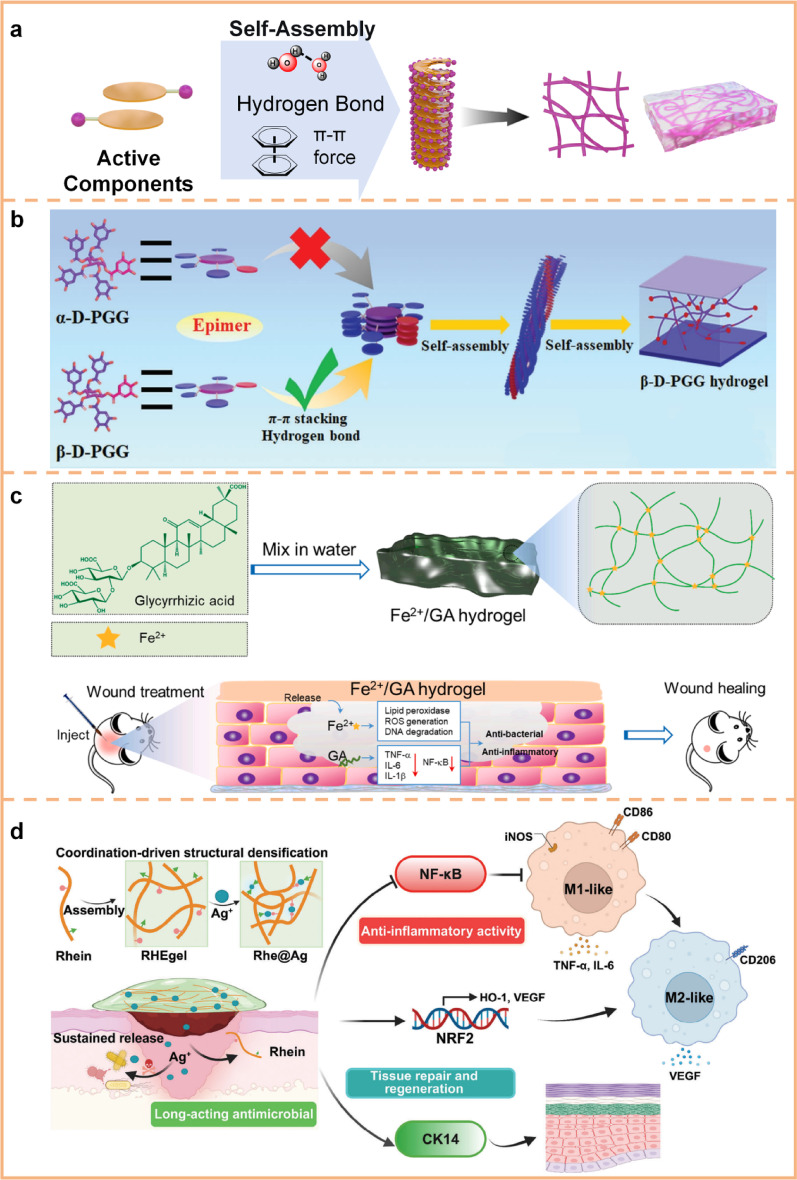
Structure and function of single-component self-assembled hydrogels of TCM. (**a**) Self-assembly mode based on intermolecular forces. (**b**) Supramolecular assembly of β-D-PGG [33]. Copyright 2024, Wiley. (**c**) Preparation of glycyrrhizic acid/Fe^2+^ coordination hydrogel and its antioxidant and anti-inflammatory mechanisms [34]. Copyright 2021, Elsevier. (**d**) The synergistic antibacterial and immunomodulatory effects of the rheic acid/silver coordination system [37]. Copyright 2024, Wiley

As shown in Fig. 3a, amphiphilic TCM components such as glycyrrhizic acid and rhein can spontaneously form supramolecular hydrogels (Table 1), which often exhibit temperature sensitivity [33–39]. For example, 1,2,3,4,6-penta-O-galloyl-β-D-glucose (β-D-PGG) self-assembles into a three-dimensional network through hydrogen bonds and π–π interactions between its uniformly distributed aromatic rings and phenolic hydroxyl groups (Fig. 3b). This network acts as a physical barrier, maintains a moist wound microenvironment, and promotes cell migration and tissue regeneration [33].

**Table 1:** Representative self-assembling single-component TCM-based hydrogels

Active components	Structures	Co-assembled	Refs.
Tannin		β-D-PGG	[33]
Glycyrrhizic acid		FeCl_2_	[34]
		CMCS	[35]
Rhein		Rhein	[36, 37]
Zingibroside R1		Zingibroside R1	[38]
Mangiferin		Mangiferin	[39]

However, high concentrations of glycyrrhizic acid pose a risk of cytotoxicity. Xu et al. developed a glycyrrhizic acid hydrogel coordinated with Fe^2+^. Fe^2+^ crosslinks with the carboxyl and hydroxyl groups of glycyrrhizic acid to achieve gelation at a low concentration, avoiding cytotoxicity while enabling sustained release of two components (Fig. 3c). Glycyrrhizic acid promotes fibroblast migration and proliferation, inhibits the NF-κB pathway to exert anti-inflammatory and antibacterial effects. Fe^2+^ broadens the antibacterial spectrum, synergistically promoting wound repair [34]. Alternatively, a double-network hydrogel is constructed by combining the glycyrrhizic acid self-assembled hydrogel with carboxymethyl chitosan (CMCS) via Schiff base bonds. This combination not only enables sustained drug release but also enhances hydrogel adhesion while synergistically providing antibacterial, anti-inflammatory, and collagen-promoting effects [35].

Rhein can self-assemble into a nanofibrous network hydrogel through π–π stacking between anthraquinone rings and hydrogen bonding between phenolic hydroxyl and carboxyl groups, which targets inhibition of the TLR4/NF-κB pathway. It exhibits better sustained release performance, biocompatibility, and cellular uptake efficiency compared with free rhein [36]. As shown in Fig. 3d, Liang et al. introduced Ag^+^ to coordinate with rhein, forming a double-network hydrogel with significantly improved mechanical stability and pH-responsive release characteristics. Ag^+^ release is accelerated in acidic infected wounds and sustained in a neutral environment, prolonging the duration of action [37].

### Single-component TCM-based hydrogels utilizing polymers

#### Single-component TCM-based hydrogels via direct physical loading of TCM monomers into polymers

**Fig. 4:**
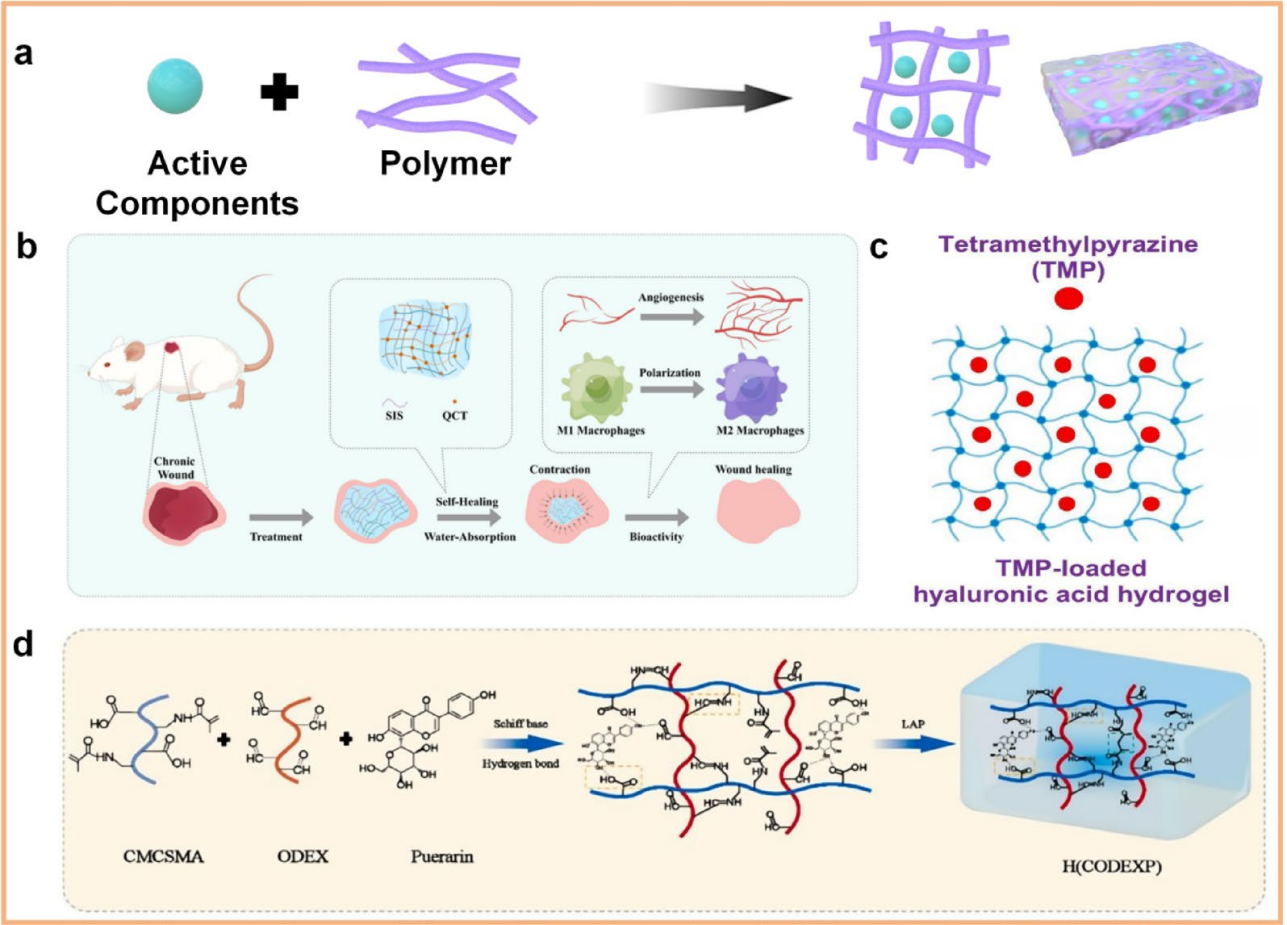
Single-component TCM-based hydrogels fabricated via physical loading into polymer networks: Schematic illustration and functional case studies. (**a**) Diagram of the physical loading strategy relying on non-covalent interactions. (**b**) QCT-loaded SIS hydrogel for immunomodulation and angiogenesis in diabetic wound healing [40]. Copyright 2023, MDPI. (**c**) TMP-loaded hyaluronic acid hydrogel facilitating macrophage polarization and wound repair [42]. Copyright 2023, Elsevier. (**d**) PUE-incorporated double-network hydrogel (H(CODEXP)) with enhanced mechanical and antibacterial properties for treating infected wounds [43]. Copyright 2025, Elsevier

Most single-component TCM hydrogels are formed by integrating TCM components into traditional hydrogel polymer matrices, with integration methods including physical loading, carrier-mediated delivery, coordination interaction, and covalent grafting. As shown in Fig. 4a, physical loading is the most straightforward strategy. It primarily relies on non-covalent interactions such as hydrophobic interactions, and hydrogen interaction to load TCM components into the polymer network. This method features mild conditions and simple operation, making it applicable to most TCM components (Table 2) [40–53].

**Table 2:** Single-component TCM-based hydrogels integrated with polymer matrices

Active components	Structures	Polymer	Ref.
QCT		The small intestinal submucosa(SIS)	[40]
		Amniotic membrane	[41]
TMP		Hyaluronic acid	[42]
Pue		CS、oxidized dextran	[43]
		CS	[44, 45]
Glucosyloxybenzyl 2-isobutylmalates extract		Carbome, Glycerol, Triethanolamine	[46]
GA		Sodium alginate, PVA, acrylic acid	[47]
Cur		Poloxamer	[48]
		CS, PVA	[49]
		Alginate	[50]
Ginsenoside Rg1		Methacryloyl hyaluronic acid, acryloyl-6-aminocaproic acid	[51]
Paeoniflorin		Hyaluronic acid	[52]
Tannin		CS, poly(ethylene glycol)	[53]

Flavonoid monomers from TCM, such as quercetin (QCT), tetramethylpyrazine (TMP), and puerarin (Pue), generally can drive the polarization of macrophages in wounds from the pro-inflammatory M1 phenotype to the reparative M2 phenotype, thereby exerting antioxidant, anti-inflammatory, and immunomodulatory activities. They can be loaded into different hydrogel matrices via non-covalent interactions including hydrogen bonds and hydrophobic interactions to exert synergistic effects. For example, loading QCT, TMP, and Pue into SIS hydrogels (Fig. 4b) [40], hyaluronic acid hydrogels (Fig. 4c) [42], H(CODEXP) hydrogels (Fig. 4d) [43], and chitosan (CS) hydrogels [44, 45], respectively, can improve the stability and bioavailability of these poorly soluble flavonoid monomers. The hydrogel matrix not only serves as a delivery carrier but also synergizes with the efficacy of flavonoid monomers through its structural characteristics (such as the biological activity of SIS, the antibacterial property of CS, and the mechanical enhancement of the H(CODEXP) double network) to jointly optimize the wound healing microenvironment.

However, physical loading systems have notable limitations. The weak interactions between drugs and carriers often lead to burst drug release. The poor controllability of release kinetics makes it difficult to adapt to the dynamic changes of the wound microenvironment. Besides, this strategy has limited ability to improve the mechanical properties of hydrogels, and may even compromise the integrity of the hydrogel network structure.

#### Single-component TCM-based hydrogels via carrier-mediated loading of TCM monomers

**Fig. 5:**
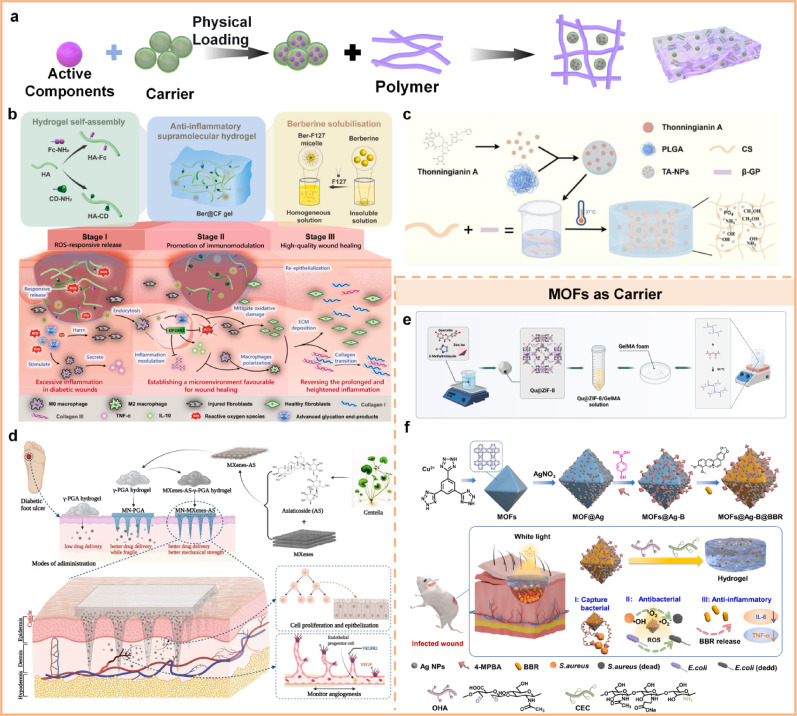
Structure and function of TCM-based hydrogels utilizing carrier-loaded TCM monomers. (**a**) Assembly strategy for single-component TCM-based hydrogels using carrier-loaded TCM monomers. (**b**) The ROS-responsive ferrocene-cyclodextrin supramolecular hydrogel system (Ber@CF gel) developed by Liang et al. [54]. Copyright 2025, Elsevier. (**c**) The TA-NPs@Gel developed by Yun et al. [56]. Copyright 2025, Elsevier. (**d**) The MXene-enhanced MN hydrogel system designed by Wang et al. achieves deep delivery and sustained release of AS, promoting fibroblast proliferation and vascular regeneration [57]. Copyright 2022, Springer Nature. (e) The ZIF-8 loaded QCT composite hydrogel constructed by Chen et al. synergistically exerts anti-inflammatory, antibacterial, and pro-angiogenic effects through sustained release of QCT and zinc ions [58]. Copyright 2024, MDPI. (**f**) The multifunctional composite hydrogel based on MOFs@Ag-B@BBR integrates bacterial targeting, photocatalytic sterilization, and sustained drug release for photothermal-chem synergistic therapy of infected wounds [55]. Copyright 2024, Elsevier

As a functional drug-carrying nanocarrier, the nanomedicine delivery system can enhance the bioavailability of therapeutic compounds and achieve targeted delivery and controllable release. As illustrated in Fig. 5a, loading TCM monomers into nanocarriers prior to assembly with polymer hydrogel matrices can overcome the limitations of conventional systems in achieving sequential and controlled drug release. This strategy simultaneously enhances the aqueous solubility and transdermal permeability of TCM compounds, optimizes their pharmacokinetic profiles, reduces potential toxicity, and increases drug accumulation and penetration at the wound site. By designing carriers with functions such as antibacterial and environmental response, multi-functional synergy can also be achieved with TCM monomers, thereby accelerating wound healing [54–60].

The level of reactive oxygen species (ROS) in diabetic wounds is 3 to 5 times higher than that in normal tissues, which not only promotes the polarization of M1 macrophages but also continuously induces chronic inflammatory responses. Responsive intelligent delivery systems are designed based on the pathological microenvironment characteristics of complex wounds (e.g., high ROS, low pH). Such systems aim to optimize the efficacy of TCM components and enable multi-stage regulation of diabetic wounds. Liang et al. [54] constructed a ROS-responsive supramolecular hydrogel (Ber@CF gel). Ber was encapsulated in Pluronic F127 micelles and then embedded in a hyaluronic acid matrix. In high-ROS diabetic wounds, gel disintegration triggered release, and Ber enhanced anti-inflammatory and pro-healing effects by inhibiting EIF2AK2, scavenging ROS, and promoting macrophage M2 polarization (Fig. 5b). Yun et al. [56] developed a thermosensitive CS hydrogel (TA-NPs@Gel). Magnolol A was delivered by Poly(lactic-co-glycolic acid) nanoparticles to reduce oxidative damage and promote angiogenesis and combined with the antibacterial properties of CS to accelerate wound closure (Fig. 5c). Zhang et al. [61] further designed a pH-responsive hydrogel. Polydopamine nanoparticles co-loaded with silver nanoparticles and curcumin (PDA@Ag&Cur NPs) were mixed with vascular endothelial growth factor (VEGF). Sequential release of antibacterial and anti-inflammatory effects was achieved through dynamic Schiff base crosslinking to meet the needs of multi-stage regulation of diabetic wounds.

Microneedle (MN) can penetrate the stratum corneum for deep delivery of TCM components, making them suitable for chronic wound repair. MXenes is transition metal carbide/nitride. Wang et al. designed a MXene-reinforced MN hydrogel system (MN-MXenes-AS): asiaticoside (AS) was loaded into a poly-γ-glutamic acid (γ-PGA) hydrogel reinforced with Ti_3_C_2_ MXenes, injected into a MN mold, and dried to form; MXenes enhanced mechanical strength to penetrate the stratum corneum, and γ-PGA rapidly dissolved to release AS, which significantly promoted diabetic wound healing by promoting fibroblast proliferation, epithelial regeneration, and angiogenesis (Fig. 5d).

Metal-organic frameworks (MOFs) are ideal drug carriers due to their high specific surface area, tunable pore structure, and functional sites. They can exert antibacterial effects by releasing metal ions or generating ROS in response to light [62–81]. Gelatin methacryloyl (GelMA) is also a commonly used matrix for hydrogel dressings. Chen et al. [58] constructed a Qu@ZIF8/GelMA hydrogel. QCT was loaded into ZIF-8 nanoparticles and then embedded in an injectable GelMA hydrogel, which continuously released QCT and Zn^2+^. Zn^2+^ had antibacterial and pro-angiogenic effects, and synergized with QCT (Fig. 5e). Our research group loaded Ber into silver-modified MOFs (MOFs@AgB@BBR) and successfully prepared a multifunctional composite hydrogel for infected wound treatment (Fig. 5f) 55]. Silver nanoparticles endowed photocatalytic ROS generation ability and provided boronic acid groups for bacterial targeting. Ber was sustainably released to exert anti-inflammatory effects. This system efficiently killed *S. aureus* and MRSA under visible light, while eliminating bacteria, reducing inflammation, and promoting tissue regeneration.

#### Single-component TCM-based hydrogels via coordination interactions

**Fig. 6:**
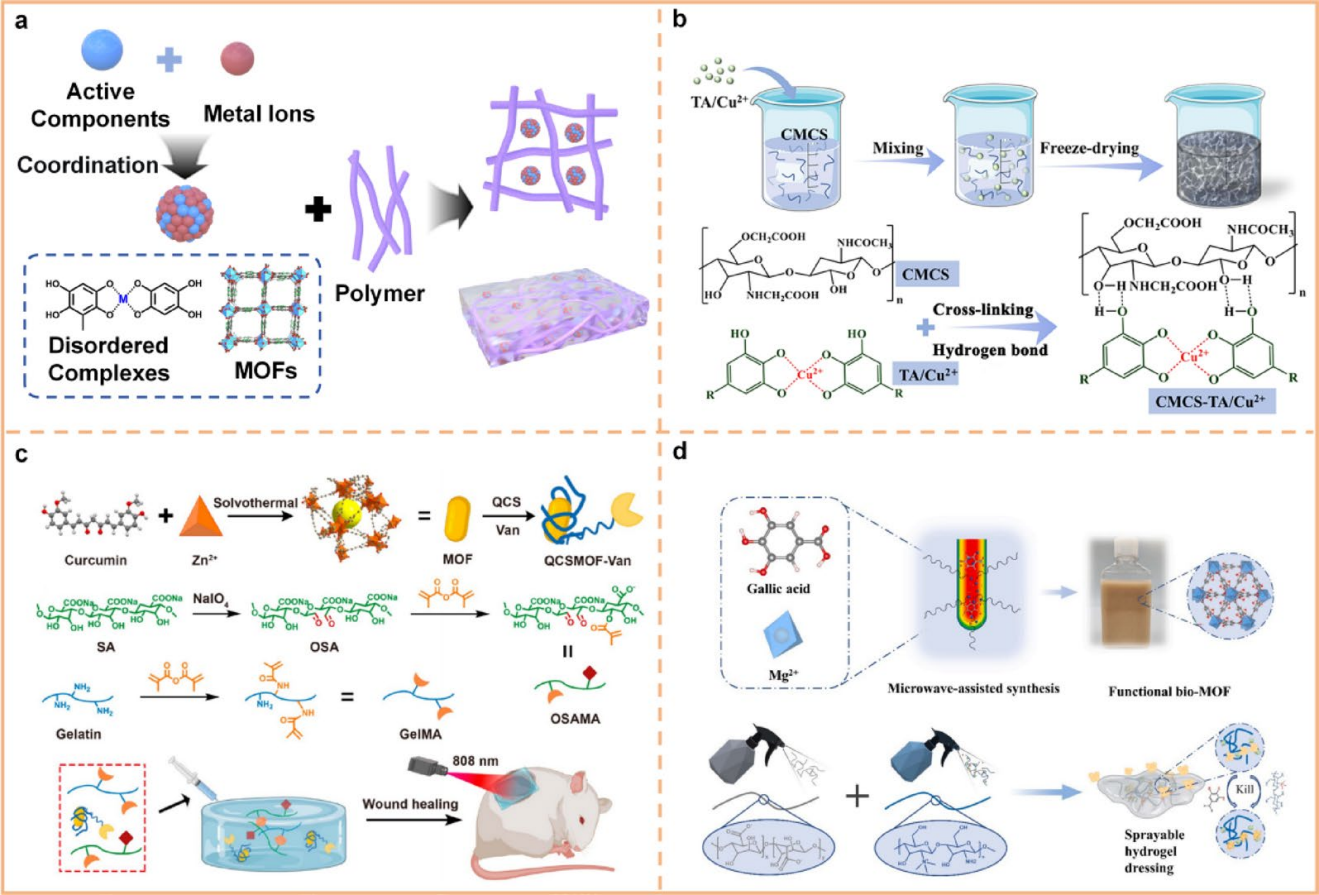
Structure and function of single-component TCM-based hydrogels via coordination interactions. (**a**) Schematic diagram of the ternary coordination assembly involving TCM monomer, metal ion, and polymer. (**b**) Microwave-assisted synthesis and structure of the Tannic Acid/Cu^2+^-CMCS hydrogel [82]. Copyright 2024, Dovepress. (**c**) Solvothermal synthesis of the Cur-Zn^2+^ MOF and its application within a GelMA hydrogel [83]. Copyright 2022, ACS. (**d**) Sprayable Gel/MOF hydrogel incorporating Mg^2+^-GA MOF nanoparticles, providing rapid gelation, oxidative stress mitigation, and pro-regenerative ion release for diabetic wound management [85]. Copyright 2024, Elsevier

Coordination bonding provides a more precise regulatory approach for TCM-based hydrogels. As shown in Fig. 6a, polyphenolic TCM components such as tannic acid (TA) and GA, due to their abundant catechol/pyrogallol structures, can form stable coordination complexes or even crystalline porous MOFs with various metal ions (e.g., Fe^3+^, Cu^2+^, Zn^2+^) [82–86].

Huang et al. [82] utilized the ortho-phenolic hydroxyl groups of TA to coordinate with Cu^2+^ and form TA/Cu^2+^ nanoparticles, which were dispersed in a CMCS matrix to construct a three-dimensional porous network with physical–chemical dual crosslinking via freeze-drying (Fig. 6b). The polyphenolic structure of TA endows it with free radical scavenging ability, and its coordination stabilization prolongs the anti-inflammatory effect. Metal ions (such as Cu^2+^) can promote angiogenesis by regulating the expression of VEGF and matrix metalloproteinases. Additionally, Cu^2+^ serves as a coordination center to stabilize network structures. The carboxyl and amino groups of CMCS form hydrogen bonds and electrostatic interactions. These interactions enhance mechanical support and biocompatibility. The synergy of the three components enables the hydrogel to possess high mechanical stability, sustained release properties, anti-inflammatory, pro-angiogenic, and scar-reducing effects.

Furthermore, MOFs are considered promising structures for next-generation wound dressings due to their ultra-high specific surface area, designable pore size, stimulus responsiveness, biocompatibility, tunable active sites, and modifiable functional groups [87]. As shown in Fig. 6c, Huang et al. [83] self-assembled Cur and Zn^2+^ to form a porous MOF (QCS-MOF-Van), achieving high loading and sustained release of Zn^2+^ and vancomycin and conferring durable antibacterial activity. In this material, Cur not only possesses the immunomodulatory and anti-inflammatory effects of flavonoids but its conjugated structure also participates in coordination assembly to construct the MOF skeleton, while endowing the hydrogel with photothermal effect (heating to 43 °C under 808 nm NIR); Zn^2+^ promotes vascular and nerve regeneration and exerts rapid bactericidal effects together with vanadium ions. By embedding MOFs into a double-network hydrogel composed of GelMA and methacrylic anhydride-modified oxidized sodium alginate (OSA), cascade repair of chronic infected wounds is achieved.

To improve clinical applicability, sprayable coordination hydrogels have also been developed. As shown in Fig. 6c, Lian et al. [85] coordinated Mg^2+^ with the ortho-phenolic hydroxyl groups of GA to form Mg-GA MOF nanoparticles, which were physically embedded in a sodium alginate-hydroxypropyl trimethyl ammonium chloride chitosan (HACC) hybrid system and rapidly crosslinked into a gel via Ca^2+^-induced ionic crosslinking. The tri-phenolic hydroxyl structure of GA efficiently chelates Mg^2+^ to form stable MOFs while conferring ROS scavenging ability; the coordinated state of Mg^2+^ is released slowly in a physiological environment, mediating nerve and vascular regeneration; the quaternary ammonium salt structure of HACC provides broad-spectrum antibacterial properties, jointly regulating the microenvironment of oxidative stress and inflammation in diabetic wounds.

Metal ion type, TCM polyphenol structure, crosslinking method, and network morphology determine material properties. These factors control mechanical properties, drug release, and biological functions. Together, they meet the functional requirements of different wounds. However, the synergistic mechanism of hydrogel components lacks quantitative analysis of multi-factor interactions; although material design is adjustable, it lacks universality; and clinical translation still faces safety verification.

#### Single-component TCM-based hydrogels via covalent integration

**Fig. 7:**
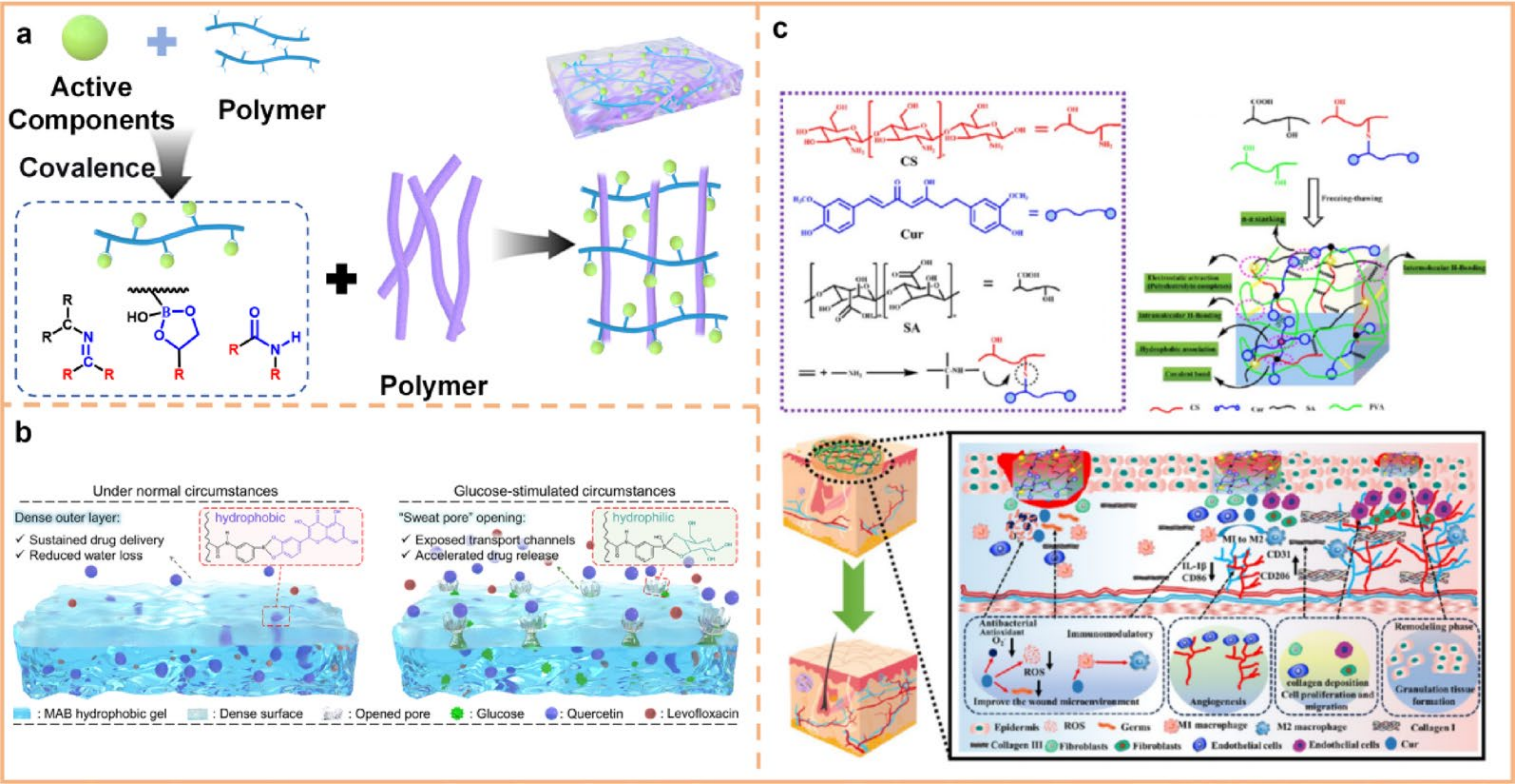
Structure and function of single-component TCM-based hydrogels via covalent integration. (**a**) Schematic diagram of network construction via covalent bonds between TCM monomers and polymers. (**b**) Construction and release mechanism of the glucose-responsive myricetin-loaded hydrogel [88]. Copyright 2025, Wiley. (**c**) Construction and function of the Cur covalently crosslinked CS/alginate/PVA IPN hydrogel [89]. Copyright 2024, ACS

Compared to intermolecular interactions and coordination bonds, covalent integration can immobilize TCM components onto polymer chains through robust chemical bonds (Fig. 7a). Commonly employed reactions include amidation, Schiff base formation, and Michael addition. For instance, gallic acid can form amide bonds with the amino groups of gelatin via its carboxyl groups, while paeonol can react with polymer functional groups through its phenolic hydroxyl groups. Covalent bonds endow hydrogels with more versatile physicochemical properties. For example, dynamic Schiff base bonds can confer self-healing capability, pH responsiveness, and sustained release functionality to hydrogels, enabling them to better adapt to wound shape and deformation, resist rupture or detachment, improve drug release controllability, while maintaining good biocompatibility [88–93].

Inspired by skin structure, a novel hydrophobic hydrogel (QL@MAB) composed of methyl acrylate (MA) and (3-acrylamidophenyl)boronic acid network was designed, while also loaded with quercetin (Q) and levofloxacin (Fig. 7b). The dense epidermal layer formed by MA ensures sustained drug release and water retention. The catechol structure of Q forms reversible boronate ester bonds with the phenylboronic acid groups of (3-acrylamidophenyl)boronic acid, which break in a high-glucose microenvironment to achieve on-demand release of Q and effectively enhance antioxidant efficiency. DPPH scavenging assays showed that Q@MAB and QL@MAB containing Q exhibited much higher efficiency than Q-free MAB and L@MAB, highlighting the critical role of Q in radical scavenging and wound healing promotion [88].

As shown in Fig. 7c, Fan et al. [89] constructed Cur interpenetrating network (IPN) hydrogels (PAVa-CSA). Cur was premixed with CS and sodium alginate (SA) to form CS/Cur-SA (CSA) pregel, and then PVA-CSA IPN hydrogel was constructed by freeze-thaw cycles with PVA. Cur not only has immunomodulatory and antiinflammatory effects, but also exerts spectral antibacterial effects through sustained-release, and upregulates CD31 expression to accelerate angiogenesis. SA regulates dispersibility, and the PVA crystalline region provides a sustained-release skeleton, ensuring that Cur exerts multi-target synergistic effects temporally in the diabetic wound microenvironment. In another example, the hydroxyl groups (-OH) on Bletilla striata polysaccharide (BSP) chains were modified with isocyanate groups (-NCO) from ureidopyrimidinone-hexamethylene diisocyanate, providing a structural basis for the hydrogel [90].

The covalent integration strategy fixes TCM components to the polymer skeleton through dynamic covalent bonds, significantly enhancing drug stability. The drug release mainly relies on material degradation rather than simple diffusion, thus achieving true long-acting sustained-release. The type of covalent bond determines the response behavior, while the network topology regulates mechanical properties and drug release kinetics. At the same time, the inherent functional groups of the monomers of TCM both participate in bonding and undertake therapeutic functions such as antioxidation and immunomodulation. This trinity design paradigm of chemical bonds - network structure - pharmacological activity provides a controllable platform for constructing intelligent responsive TCM hydrogel dressings. However, the covalent binding synthesis process is relatively complex, requiring precise control of reaction conditions, and the response efficiency and long-term stability of dynamic covalent bonds in complex in vivo environments remain unclear. The compatibility of degradation rates with tissue regeneration cycles needs to be considered.

The above systematic analysis of single-component TCM hydrogel assembly strategies is further supported and validated by the comparative data in Tables 3 and 4. As shown in Table 3, we conducted a head-to-head comparison of gelatin-based hydrogels with and without TCM components under identical evaluation metrics, which directly substantiates the unique advantages of TCM-based hydrogels. Unlike non-TCM hydrogels that rely on exogenous bioactive carriers for indirect anti-inflammatory and therapeutic effects, TCM components endow hydrogels with intrinsic, direct multi-target pharmacological activities. TCM monomers such as GA and curcumin directly inhibit the core inflammatory pathway NF-κB, while participating in the construction of the hydrogel network through their phenolic hydroxyl, carboxyl and other functional groups. This dual function enables TCM hydrogels to achieve a faster wound closure rate and higher-quality tissue repair than non-TCM hydrogels, even without additional antibiotic or growth factor loading.

Table 4 systematically compares the differences in physicochemical properties between small-molecule TCM self-assembled hydrogels and polymer-loaded TCM monomer hydrogels, which elaborates in detail how TCM components regulate the structure, rheological and mechanical properties of hydrogels. For small-molecule self-assembled systems, TCM monomers are the only building blocks of the 3D network: the number of phenolic hydroxyl groups, aromatic ring structure and hydrophilic/hydrophobic ratio of TCM molecules directly determine the strength of hydrogen bonding and π-π stacking, which in turn governs the hydrogel’s mechanical strength, viscoelasticity and rheological stability. For example, mangiferin self-assembled hydrogel has a G’ ~17 times higher than G”, showing excellent elasticity dominated by the rigid xanthone structure and abundant phenolic hydroxyl groups of mangiferin. For polymer-loaded systems, TCM components not only provide pharmacological activity, but also act as crosslinking nodes to enhance the hydrogel network. GA and dopamine form multiple hydrogen bonds with the polymer backbone, increasing the hydrogel’s G′ to 2 × 10^4^ Pa and tissue adhesion strength to 17 kPa, which is far superior to the blank polymer hydrogel.

Through the comparative analysis of Tables 3 and 4, we further identify the current limitations and knowledge gaps of single-component TCM hydrogels. Firstly, small-molecule self-assembled hydrogels have excellent biocompatibility but insufficient mechanical strength, which cannot adapt to dynamic wound environments such as joint sites. Secondly, polymer-loaded systems have adjustable mechanical properties, but most of them use simple physical loading, leading to burst release of TCM components. Most studies only focus on the therapeutic effect of single-component systems, but lack systematic comparison of structure-activity relationships between different TCM components. These gaps are exactly the core direction of the evolution from single-component to multi-component TCM hydrogel systems, which also echoes the core logic of our proposed “component-number hierarchy” framework.

**Table 3:** Comparison of therapeutic efficacy between gelatin-based hydrogels with and without TCM components

Active components	Ssembly strategy	Antibacterial functional elements	Wound healing performance	Anti-inflammatory mechanism	Scar regulation effect	Ref.
Without TCM components	Photo-crosslinking, physical entanglement	None	Full healing in 10–12 days; indirect therapeutic effect relying on exogenous bioactive carriers	Indirect anti-inflammation via M2 macrophage polarization regulated by the TLR2/PI3K/Akt pathway	Promotes angiogenesis and hair follicle regeneration, no targeted collagen regulation	[94, 95]
With TCM components	Metal coordination	GA, Zn^2+^	Full healing within 11–14 days; 91% wound closure at day 7	Direct inhibition of IL-1β, TNF-α and NF-κB pathway by GA	Ordered collagen arrangement, significant type I collagen deposition	
	Hydrogen bonding, π-π stacking	Cur, vancomycin	Full healing within 12–15 days; 89% wound closure at day 7	Direct inhibition of NF-κB pathway by Cur, promoting M1 to M2 macrophage transition	Optimized collagen I/III ratio, reduced scar formation	[34, 83, 96–98]
	Amide covalent bonding	GA	Full healing within 10–13 days; 93% wound closure at day 7	ROS scavenging and pro-inflammatory factor inhibition by GA	Enhanced collagen deposition, intact dermal structure	

**Table 4:** Comparison of physicochemical properties between small-molecule TCM self-assembled hydrogels and polymer-loaded TCM monomer hydrogels

Hydrogel category	TCM active ingredients	Mechanical strength	Stability	Viscoelasticity	Rheological properties	Toughness	Ref.
Small-molecule TCM self-assembled hydrogels	Rhein	Young’s modulus: 66.85 kPa	pH-responsive release, structurally stable	G′ is much higher than G″, elasticity-dominated	G′ and G″ remain stable in frequency/time scanning, no obvious decay	Double network + Ag^+^ coordination, excellent energy dissipation capacity	[37]
Small-molecule TCM self-assembled hydrogels	Cur	G′ remains stable at low strain, moderate mechanical strength	G′ fully recovered after multiple high/low strain cycles	G′ > G″ at low strain, typical elastic behavior	G′ fully recovers after 500% strain treatment	Ordered network structure, moderate toughness	[38]
Small-molecule TCM self-assembled hydrogels	Mangiferin	G′ is ~17 times higher than G″, excellent mechanical strength	Stable at pH 7–9, structural damage beyond this range	G′ is ~17 times of G″, excellent elasticity	Gel-sol transition occurs at 28.7% strain	High elasticity + intrinsic self-healing property	[39]
Polymer-loaded TCM monomer hydrogels	Luteolin	G′ ≈ 2244 Pa; compressive stress: 3.4 kPa @50% strain	pH-responsive Cu^2+^ release; G′ drops > 50% under acidic conditions	G′ > G″, double network enhanced viscoelasticity	Shear-thinning behavior, injectable, rheological properties adjustable for smart dressings	Double network +coordination crosslinking, excellent toughness, no fracture at 50% compressive strain	[97]
Polymer-loaded TCM monomer hydrogels	GA, dopamine	G′ ≈ 2 × 10^4^ Pa; adhesion strength ≈ 17 kPa (porcine skin)	7-day degradation rate 80–90%; swelling equilibrium time: 16 h	G′> G″, elasticity-dominated; structure destroyed at 100% strain, fully recovered at 1% strain	Rapid recovery in multiple gel-sol transition cycles at 50% strain	Hydrogen bond + physical crosslinking, good toughness and strong energy dissipation capacity	[104]
Polymer-loaded TCM monomer hydrogels	Scutellaria TF	G′ ≈ 2 × 10^4^ Pa; G′ higher than blank polymer hydrogel	Micro-nano porous structure, high swelling rate, slow degradation, sustained release over 7 days	G′> G″, chiral structure enhances elasticity	Enhanced shear thinning after multiple freeze-thaw cycles, injectable	Chiral structure + polymer network, enhanced toughness and tear resistance	[105]

In summary, single-component systems serve as indispensable fundamental research models for elucidating the assembly behavior, release mechanisms, and biological effects of TCM ingredients, providing clear structure-activity relationship information that lays the groundwork for understanding more complex systems. However, current systems still focus on single or dual components, whose single pharmacological activities often fail to meet the multifaceted therapeutic demands of chronic wound treatment and have not yet truly embodied the holistic regulatory concept of “multi-component, multi-target” inherent to TCM formulas. This limitation somewhat restricts their clinical application prospects and drives research towards the development of more complex multi-component systems.

## Dual-component TCM-based hydrogels

Wound healing is a dynamic process involving multiple overlapping stages of hemostasis, inflammation, proliferation, and remodeling. Hydrogels loaded with a single component struggle to exert simultaneous effects across these different healing phases and provide comprehensive intervention. As the second progressive level of the component-number hierarchy framework (Fig. 1), dual-component TCM-based hydrogels achieve the first synergistic design of TCM components, which corresponds to the “Emperor-Minister” compatibility logic in classic TCM theory. These systems overcome the functional limitations of single-component systems through rational synergistic assembly, achieving functional integration and spatiotemporal regulation of wound healing. Dual-component TCM-based hydrogel systems represent a significant shift from simple loading to synergistic design, serving as a critical bridge connecting fundamental research and clinical application. At this hierarchical level, synergistic effects arise from meticulously designed interactions between the two active components, or between an active component and a functional material, enabling functional enhancement and intelligent upgrades.

### Design principles and assembly modes of dual-component TCM-based hydrogels

**Fig. 8:**
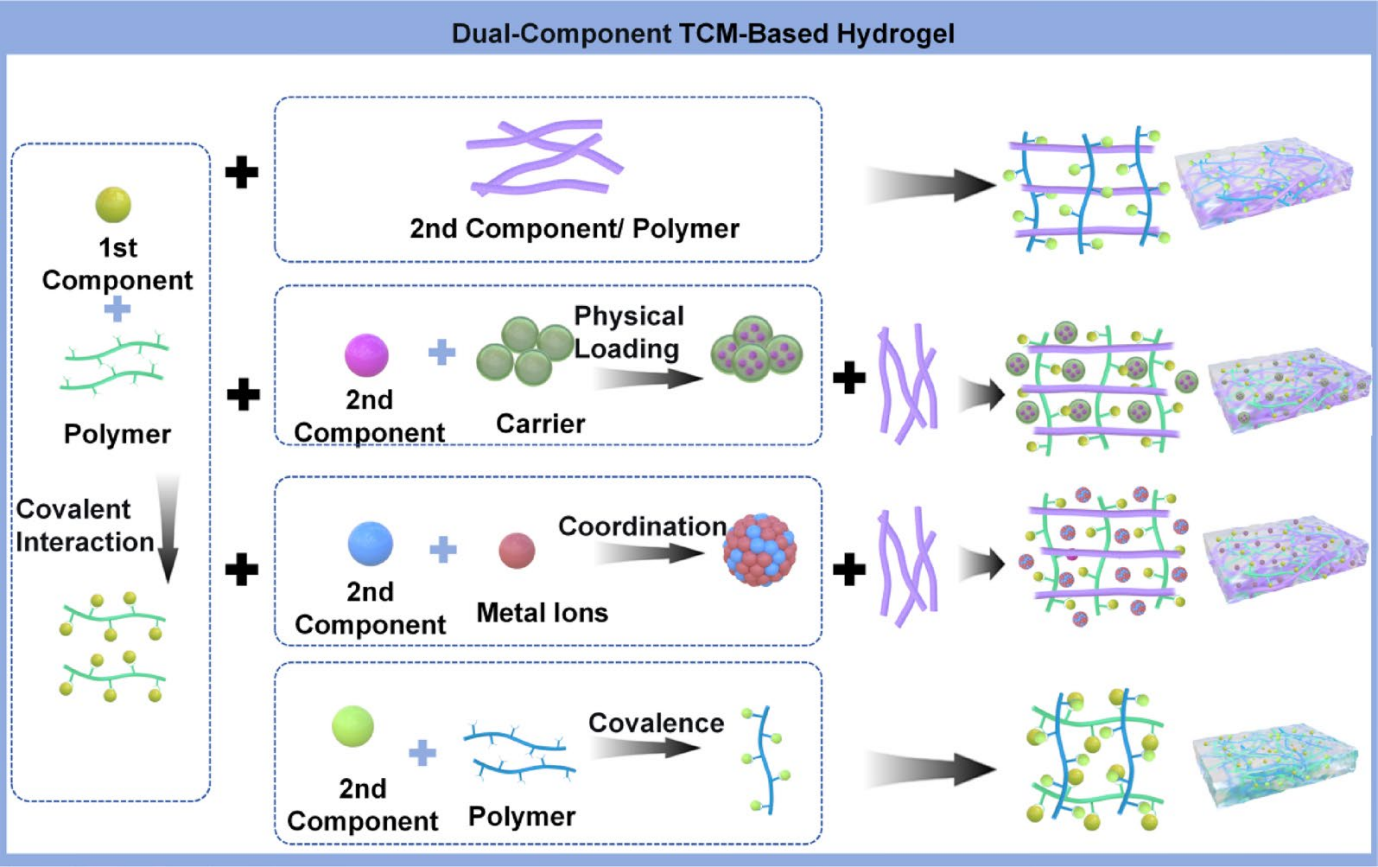
Synergistic assembly modes of dual-component TCM-based hydrogels

As illustrated in Fig. 8, the assembly of dual-component TCM-based hydrogels demonstrates a systematic construction logic. A typical strategy involves the first component forming a covalent bond with the polymer, followed by the introduction of the second component through various synergistic pathways such as physical loading, coordination, or covalent bonding (Table 5) [99–101, 106–110]. This evolution not only enhances the functional integration level of the materials but, more importantly, achieves precise intervention in different stages of the wound healing process, providing innovative material solutions for the modernization of TCM.

**Table 5:** Representative self-assembling dual-component TCM-basedhydrogels

1stComponent	Structures	2ndComponent	Structures	Polymer	Ref.
FA		BSP		Methacrylate-modified chitosan	[107]
GA		BSP		CS, Fe^3+^	[108]
CA		Glabridin		CS, poly(N-isopropylacrylamide) (PNIPAm)	[109]
Bletilla striatapolysaccharide		PPD		methacrylicanhydride	110
CA		Genipin		Zn2+, gelatin	102
BSP		Tea polyphenol		Adipic acid dihydrazide modified gelatin	[100]
Glycyrrhizic acid		AS		GelMA	[101]
Keratin	—	Protocatechuic aldehyde		Zn^2+^	[111]

**Fig. 9:**
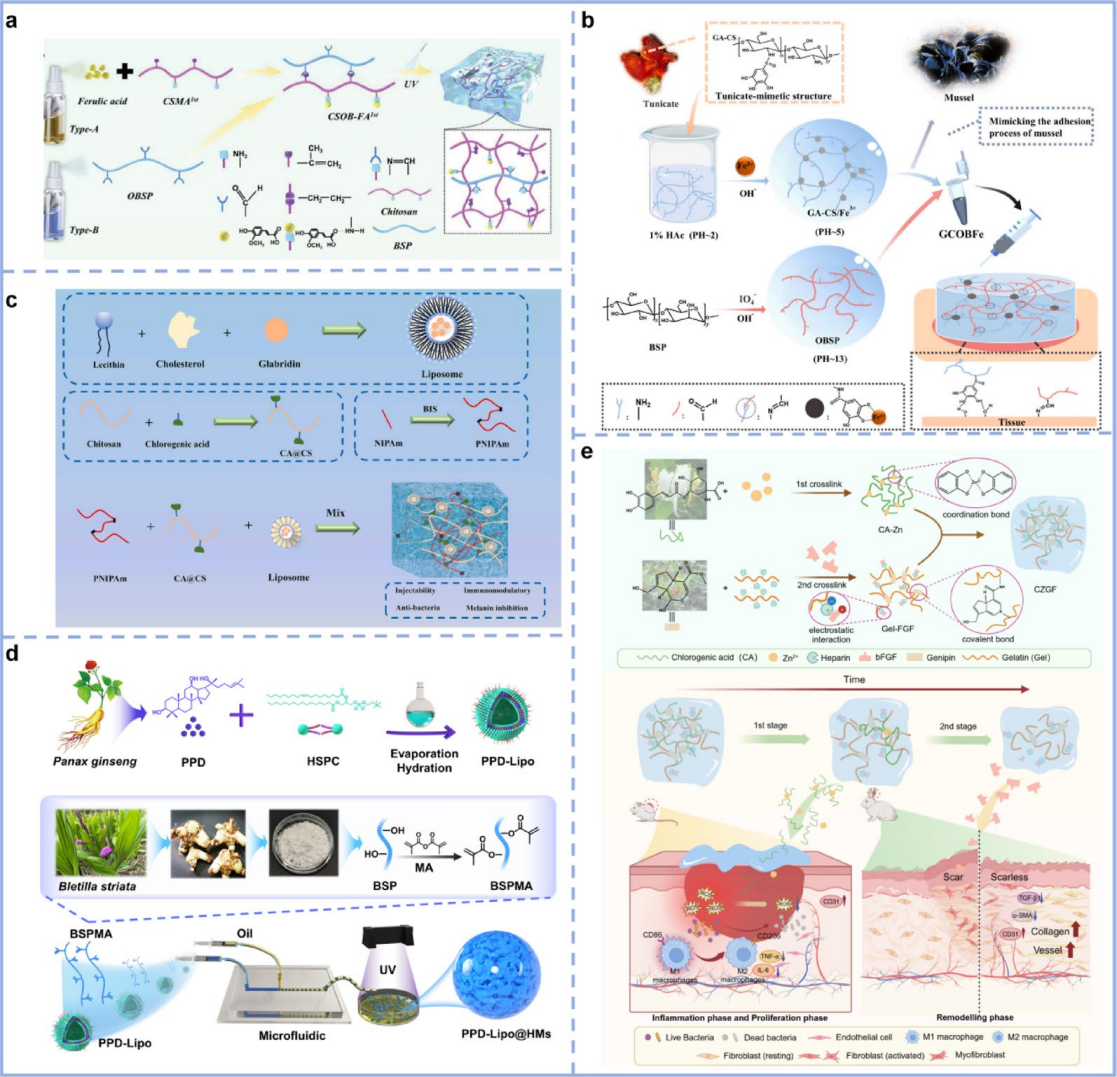
Schematic diagram of synergistic therapeutic mechanisms in dual-component TCM-based hydrogels. (**a**) Instant film-forming and immunomodulatory functions of the FA-polysaccharide sprayable hydrogel [106]. Copyright 2024, Wiley. (**b**) Photothermal antibacterial mechanism of the GA-metal coordinated dual dynamic cross-linked hydrogel [107]. Copyright 2021, Elsevier (**c**) Multi-stage drug release and pigment regulation functions of the glabridin liposome-thermosensitive hydrogel system [108]. Copyright 2025, Elsvier. (**d**) Synergistic immunomodulation and angiogenesis of the protopanaxadiol liposome-microsphere system [109]. Copyright 2024, Springer Nature. (**e**) Coordination pre-assembled nanounit integration strategy: stage-specific therapeutic mechanism of the CA-Zn^2+^ coordination network and growth factor programmed release system [101]. Copyright 2025, Springer Nature

### Representative construction strategies of dual-component TCM-based hydrogels

#### Direct integration of the second active component into polymer architectures

A prominent design strategy involves integrating the second active component directly into the polymer architecture. As shown in Fig. 9a, Zhong et al. [106] developed a two-component sprayable hydrogel system. When mixed, solutions containing ferulic acid (FA) with methacrylated chitosan and oxidized *Bletilla striata* polysaccharide (OBSP) rapidly form an adaptive gel network (CSOB-FA) via dynamic Schiff base bonds. After photo-curing, the hydrogel adheres tightly to tissue, with FA retained via hydrogen bonding and π–π stacking. FA contributes antioxidant, antibacterial, and anti-inflammatory effects, while OBSP facilitates immunomodulation by driving macrophage polarization toward the M2 phenotype and supporting collagen deposition. Together, these functions enable effective management of large or irregular wounds in both emergency and chronic care scenarios.

#### Carrier-mediated loading of the second active component for spatiotemporal controlled release

In the systems shown in Fig. 9c-d, the second active component is introduced into the hydrogel via a carrier-loading strategy, achieving drug protection and controlled release. Cao et al. [108] engineered a thermosensitive double-network hydrogel for diabetic wound healing by combining a CA@CS/PNIPAm IPN with glabridin-loaded liposomes (Fig. 9c). The Lipo enable sustained glabridin release, inhibiting melanogenesis and providing antioxidant and anti-inflammatory benefits. Meanwhile, chlorogenic acid (CA) exhibits antibacterial and immunomodulatory activities and accelerates angiogenesis and tissue repair. Guo et al. [109] developed a microsphere-based TCM hydrogel system (Fig. 9d), covalently binding BSP with methacrylate anhydride (MA) to form photosensitive BSPMA, encapsulating 20(S) -propanginseng diol (PPD) in Lipo Binding with BSPMA to form nanocomposite microspheres (PPD-Lipo@HMs), these microspheres exhibit high swelling capacity and sustained PPD release, PPD activates the PI3K/Akt/mTOR pathway to promote angiogenesis, and BSP exerts immunomodulatory effects to synergically promote diabetic wound regeneration. Gong et al. [111] constructed EGCG@Fe nanoparticles via metal-polyphenol coordination self-assembly, then loaded salvianolic acid B and glucose oxidase (GOx) through non-covalent interactions, and embedded the nanoparticles into a dynamic network of quaternized chitosan (QCS) and oxidized fucoidan (OFu) crosslinked by Schiff base bonds. In this system, Epigallocatechin gallate (EGCG)’s ortho-phenolic hydroxyl groups coordinate with Fe^3+^. This gives the nanoparticles stability, pH-responsive dissociation, and ROS scavenging ability. The phenolic acid structure of salvianolic acid B contributed anti-inflammatory and pro-angiogenic activities; GOx enhanced antibacterial effects by consuming glucose and producing H_2_O_2_. The quaternary ammonium salts of QCS provided broad-spectrum antibacterial properties. And amino groups of QCS formed dynamic Schiff base bonds with the aldehyde groups of OFu, endowing the network with self-healing and microenvironment-responsive degradation capabilities. The hierarchical structure achieved sequential release of each component, synergistically realizing multi-target repair including antibacterial, antioxidant, immunomodulatory and pro-regenerative effects.

#### Pre-assembled functional nano-unit integrated dual-network systems

For even more sophisticated temporal control, pre-assembled functional nano-units can be incorporated. Lin et al. [101] designed a programmed release dual-network hydrogel (CZGF hydrogel) for scarless healing of infected diabetic wounds (Fig. 9e). A metal-phenolic network (CA-Zn) formed from CA and Zn^2+^ serves as the first network, offering broad-spectrum antibacterial and ROS-scavenging functions in the early inflammatory phase. This is combined with a second network of geniposide-crosslinked heparin-modified gelatin (Gel-FGF) that slowly releases bFGF during the proliferation-remodeling phase. This sequential release profile ensures timely antibacterial and anti-inflammatory action followed by sustained pro-regenerative signaling, effectively minimizing scar formation.

In another related study utilizing natural phenolic chemistry, Chen et al. [107] constructed a low-cost dual-dynamically crosslinked hydrogel (Fig. 9b). After grafting GA onto CS (GA-CS), a metal-polyphenol coordination network was first formed through the coordination of its ortho-phenolic hydroxyl groups with Fe^3+^, which was then crosslinked with OBSP via Schiff base bonds to form a dual dynamic network. In this system, the pyrogallol groups of GA not only mediate Fe³⁺ coordination to stabilize the network but also endow photothermal effects (rapid sterilization) and wet tissue adhesion; the cationic properties of the CS backbone and the phenolic hydroxyl groups of GA synergistically provide durable antibacterial activity; the polysaccharide backbone of OBSP regulates the network degradation rate through dynamic Schiff base bonds and enhances biological activity. The synergy of coordination bonds and Schiff base bonds enables the multifunctional integration of photothermal sterilization, tissue adhesion, and controllable degradation.

Although dual-component TCM-based hydrogels have made notable progress in functional synergy, most studies fail to rigorously distinguish and verify the additive versus synergistic effects between the two components. Furthermore, the lack of systematic optimization of component ratios and the absence of dose-effect curves make it difficult to define the optimal formulation. The design and verification of temporal synergy are disconnected, and quantitative studies on the matching between pharmacokinetics and healing stages are insufficient. Meanwhile, the complexity of the mechanism of two-component systems exceeds that of single-component systems, yet they are insufficient to reflect the overall regulatory advantages of TCM compound prescriptions, and the introduction of the second component may lead to functional redundancy. More importantly, material-pharmacological interactions are often neglected. The second component may indirectly regulate the release and effect of the first component by changing physical properties such as network structure and degradation rate, and this “physical synergy” may be mistaken for “pharmacological synergy”. Future studies must go beyond simply showing “A + B is better than single use.” They should define synergy, screen optimal ratios, and demonstrate temporal matching. In-depth analysis of material-pharmacological interactions is also needed.

## Multi-component and formula-mimicking TCM-based hydrogels

Single-component TCM-based hydrogels have established a foundational understanding of structure-activity relationships, yet their limitations are apparent due to single pharmacological activities failing to address the multi-stage demands of wound healing and lacking the holistic regulatory characteristics inherent to traditional TCM. Dual-component systems represent a critical breakthrough through synergistic design. Whether incorporating the second active component as part of the polymer network, achieving controlled release via carrier loading, or pre-assembling functional nano-units, these systems can cover more therapeutic targets through spatiotemporal controlled release and functional synergy, serving as a core bridge connecting fundamental research and clinical application. However, it is important to note that the synergistic effects in dual-component systems are still confined to interactions between two active components. As the highest and most complex level of the proposed component-number hierarchy framework (Fig. 1), multi-component and formula-mimetic TCM-based hydrogels are the core carrier to fully translate the classic TCM “EMAC” holistic compatibility theory into modern biomaterial design. Unlike lower-level systems, these systems achieve rational hierarchical assembly of multiple components with well-defined EMAC roles, to replicate the holistic multi-target synergistic effects of TCM formulas in chronic wound treatment.

**Fig. 10:**
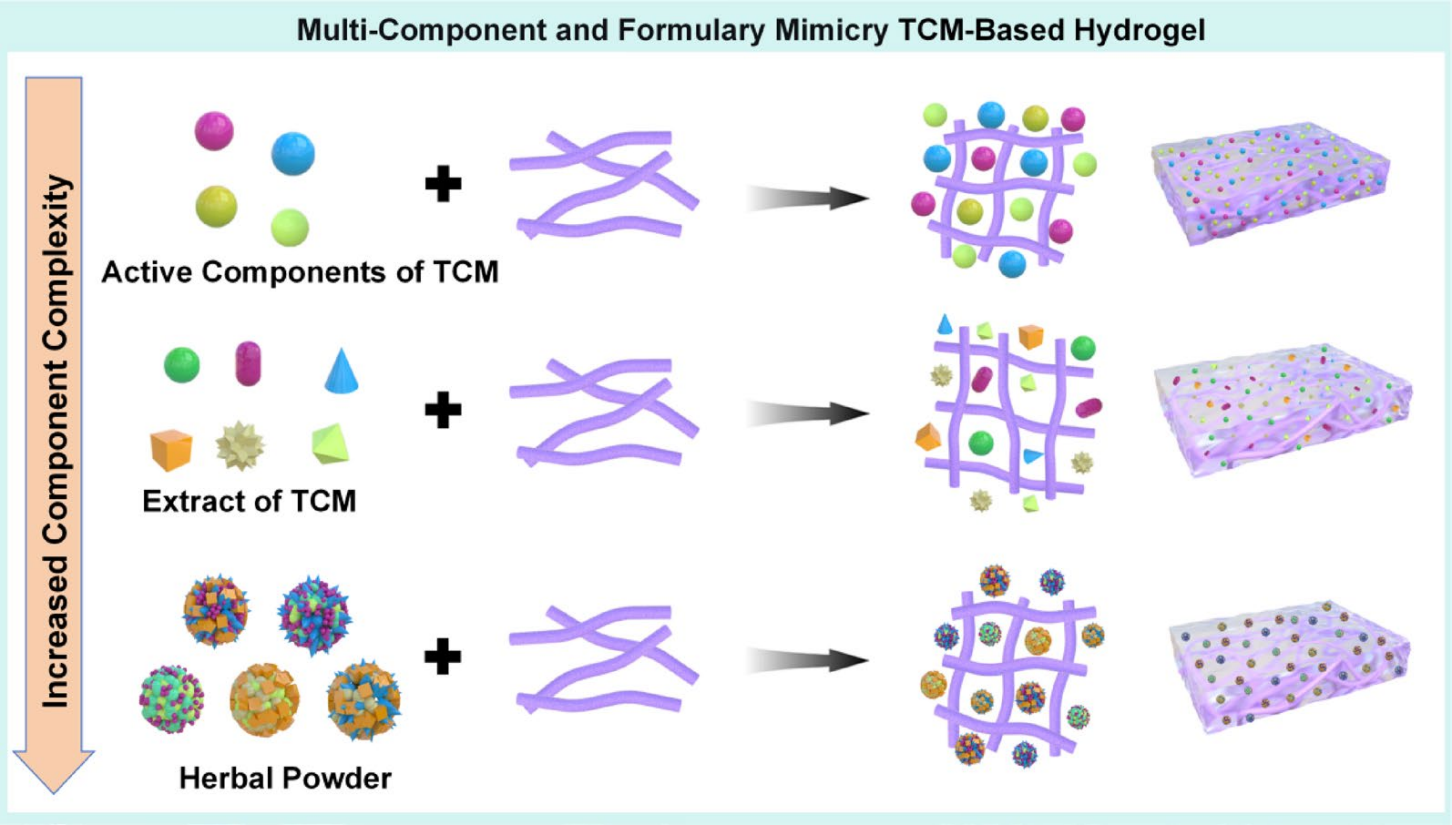
Schematic diagram of synergistic therapeutic mechanisms in multi-component and formula-mimicking TCM-based hydrogels

### Current research status and core construction pathways of multi-component systems

Compared to the well-defined components and clear mechanisms of single/dual-component TCM-based hydrogels, current research on multi-component and formula-mimicking systems remains in a relatively early stage, with significantly insufficient refinement in both construction strategies and mechanistic understanding. As shown in Fig. 10, the core construction pathways for reported multi-component systems predominantly rely on simple physical blending. This includes directly mixing active TCM components with polymers, adding polymers to TCM extracts for gelation, or filling TCM powders into polymer networks. These approaches lack the sophisticated assembly design seen in dual-component systems [102, 103, 105, 117–124].

### Representative construction strategies of multi-component TCM-based hydrogels

#### Direct physical blending-based multi-component hydrogel systems

**Fig. 11:**
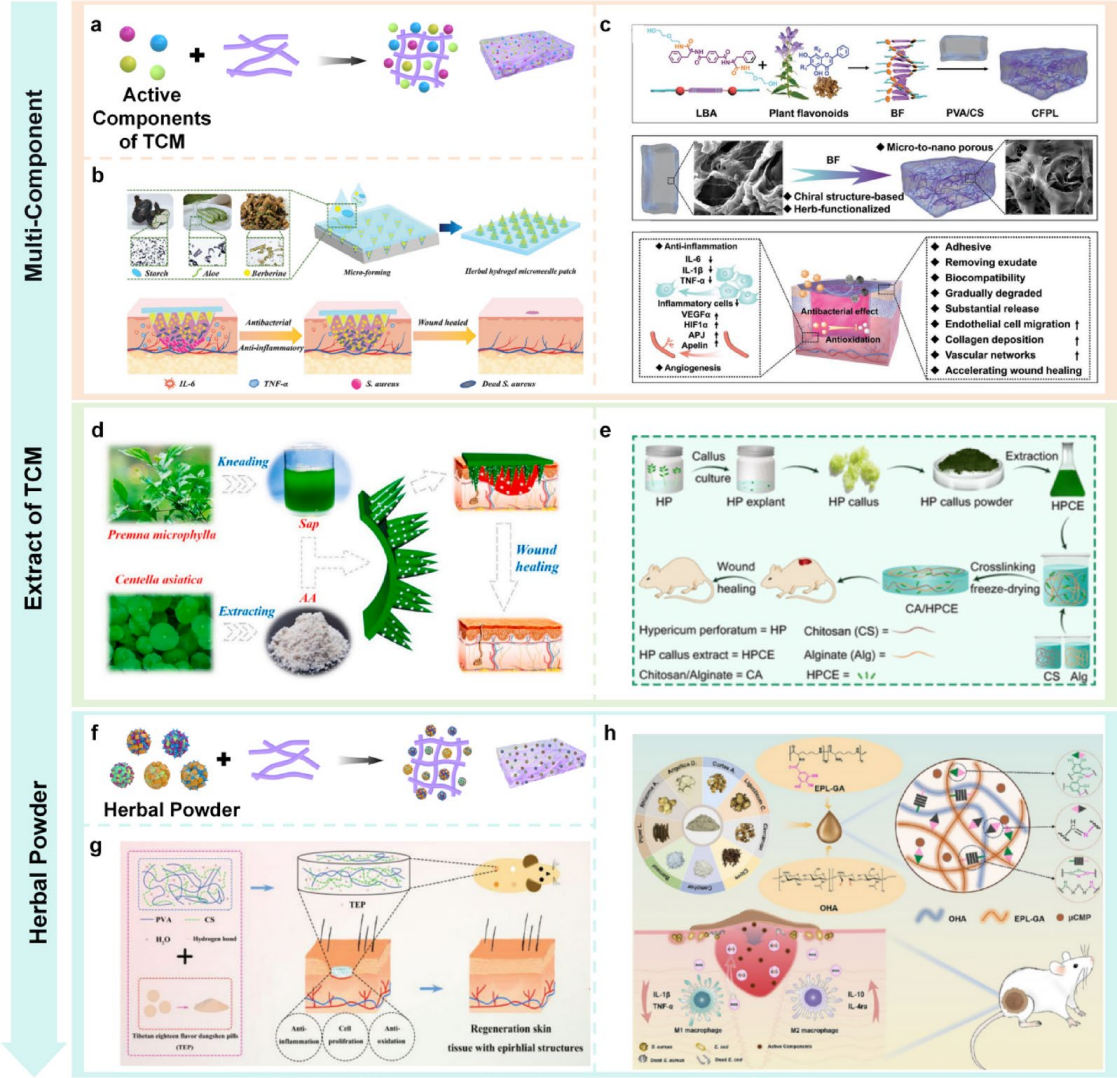
Structural and functional examples of multi-component/formula-mimicking TCM-based hydrogels. (**a**) Schematic diagram of the synergistic assembly of multiple active components. (**b**) Construction of Ber–aloe vera freeze-dried powder composite starch MN hydrogel and its application in antibacterial wound healing [119]. Copyright 2024, Wiley. (**c**) Structure and multifunctionality of the flavonoid–chiral gelator co-assembled composite PVA/CS hydrogel [105]. Copyright 2022, Wiley. (**d**) Preparation and deep delivery effect of *Premna microphylla* juice–asiaticoside composite MN hydrogel [121]. Copyright 2021, Elsevier. (**e**) Construction of the HPCE-loaded CS/alginate hydrogel and its mechanism for anti-scarring healing [117]. Copyright 2024, ACS. (**f**) Schematic diagram of the preparation of TCM powder/superfine extract-embedded hydrogel. (**g**) Diabetic wound repair effect of the TEP powder-composite PVA/CS hydrogel [118]. Copyright 2021, Royal Society of Chemistry. (h) Topical adjuvant function of the EPL-GA-TCM superfine powder composite hydrogel [122]. Copyright 2024, ACS

Direct physical blending of multiple active TCM components with polymers to form multi-component hydrogels represents the simplest strategy (Fig. 11a). Luan et al. [119] used a raw-cooked starch paste process to create a homogeneous gel from water chestnut starch, into which lyophilized aloe vera powder and Ber were incorporated (Fig. 11b). The gel was then molded into MN capable of penetrating the skin barrier, enabling deep tissue delivery where Ber exerts antibacterial and anti-inflammatory effects alongside aloe vera’s pro-angiogenic and collagen-promoting activity. Beyond simple blending, molecular co-assembly offers a pathway to enhance material properties. As shown in Fig. 11c, Sun et al. [105], based on the theory of “chiral microenvironment regulation of cell behavior,” prepared multi-flavonoid co-assemblies by non-covalently combining flavonoids with a shared skeleton and chiral gelators, which improve the solubility of flavonoids and achieve sustained release and activity protection.

#### Molecular co-assembly and hierarchical covalent integration strategies

Covalently grafting gastrodia elata polysaccharide (GEP) onto the CS backbone, a primary network was constructed via Schiff base crosslinking with oxidized gastrodia elata polysaccharide(OGEP), and EGCG microspheres were introduced as functional loads. In this system, Schiff base bonds endow the network with self-healing and microenvironment-responsive degradation properties; the ortho-phenolic hydroxyl groups of EGCG mediate antioxidant and antibacterial activities; the hydrophilic backbone of GEP synergizes with the cationic properties of CS to achieve hemostatic function; and the microsphere structure enables sustained release of EGCG. The synergy of chemical bonds, functional groups, and hierarchical structures collectively promotes angiogenesis, collagen deposition, and tissue remodeling in diabetic wounds.

Xin et al. [125] constructed a three-level assembled hydrogel system based on GEP. Firstly, gastrodin was covalently anchored to the CS backbone via free radical grafting. Its phenolic hydroxyl groups endow antioxidant activity, and the amino groups of CS mediate antibacterial effects. Subsequently, a dynamic covalent network was formed via Schiff base reaction with OGEP, endowing the hydrogel with self-healing, injectable, and controllable degradation properties. Finally, EGCG@GEL gelatin microspheres were embedded to achieve sustained release of EGCG, the ortho-phenolic hydroxyl groups of EGCG improve anti-inflammatory activity, and the hierarchical structure (microsphere-network dual barrier) achieves spatiotemporal controlled release. This system inhibits the IκBα/NF-κB pathway, reducing TNF-α and IL-1β. It also up-regulates VEGF and TGF-β1 to promote angiogenesis. In a diabetic wound model, it achieved a 93.43% healing rate within 14 days.

#### TCM extract and ultrafine powder integrated multi-component systems

Since TCM formulas often exist as liquid preparations, TCM extracts are more readily available than mixtures of multiple structurally defined TCM monomers. Chi et al. [121] developed a Chinese herbal MN patch using leaf juice extracted by a traditional twisting method, combined with plant ash from tofu production (Fig. 11d). The mixture was cross-linked ionically in MN molds and loaded with AS for deep delivery. Premna microphylla leaf juice has the effects of clearing away heat and toxic materials, reducing swelling, stopping bleeding, while AS exerts antioxidant, anti-inflammatory and antibacterial effects. The Premna microphylla leaf juice, obtained by a simple physical method, is solidified into a MN patch, which not only retains the activity of Premna microphylla leaves but also avoids side effects caused by complex chemical treatments. Zivari-Ghader et al. [117] proposed a hydrogel wound dressing fabricated from a CS/alginate scaffold loaded with HPCE (Fig. 11e). The TCM component HPCE is rich in active ingredients like flavonoids, phenolic acids, and hypericin. Through a sustained release mechanism, it exerts synergistic antioxidant, antibacterial, and anti-inflammatory effects, promotes fibroblast migration and orderly collagen deposition, and downregulates α-SMA expression to prevent hypertrophic scar formation.

In cases where active components are difficult to extract or best used in their native state, ultrafine powder integration provides a practical alternative Wang et al. [118] incorporated Tibetan eighteen-flavor dangshen pill (TEP) powder into a PVA/CS hydrogel (Fig. 11g). TEP is rich in phenolic acids and flavonoids, which sustained-release exerts antioxidant, antibacterial, anti-inflammatory and analgesic effects, promotes cell proliferation and ordered collagen deposition, and avoids side effects caused by excessive dosage when used directly. As shown in Fig. 11h, an ε-polylysine -GA covalent polymer (EPL-GA) was used to load an antibacterial and antioxidant TCM superfine powder, replacing traditional honey dressings and addressing issues like difficulty in cleaning and uneven drug distribution associated with traditional TCM topical preparations. The resulting hydrogel features injectability, self-healing properties, and conformability to the skin. EPL also contributes synergistic antibacterial effects by disrupting bacterial membranes [122].

Table 6 further compares the efficacy of chitosan-based hydrogels without TCM components, with single-component, dual-component, and multi-component TCM systems, evaluating antibacterial activity, wound healing rate, anti-inflammatory mechanism, and scar inhibition. Chitosan hydrogels without TCM components rely on the intrinsic mild antibacterial activity of chitosan or incorporated AgNPs/TiO_2_, achieving approximately 82% wound closure by day 7 and near-complete healing by day 14. Their anti-inflammatory effects are indirect and limited. Single-component TCM hydrogels, such as GA, PUE, tannic acid, luteolin, introduce direct antioxidant and anti-inflammatory activities via TCM monomers, regulating macrophage polarization and accelerating the transition from inflammatory to proliferative phase. The wound area reduces to 2.86% by day 12, with a 95% healing rate by day 14. Scar quality improves with optimized collagen I/III ratio and partial hair follicle regeneration. Dual-component TCM hydrogels achieve synergistic effects through multiple crosslinking strategies, such as Schiff base, metal-phenol coordination, amide bonds. Wounds are almost completely closed by day 13–15, with a 92% healing rate in diabetic mice by day 14. Multi-component synergy significantly downregulates multiple inflammatory factors and promotes M1-to-M2 macrophage conversion. Multi-component and formula-mimicking systems, such as HPCE extract, TEP powder, and flavonoid co-assemblies, explicitly demonstrate anti-hypertrophic scarring effects, including the inhibition of α-SMA expression. These systems also exhibit potent anti-inflammatory activity and promote ordered collagen deposition. Consequently, they accelerate re-epithelialization, angiogenesis, and high-quality tissue repair. These multi-component systems also achieve superior ECM remodeling via downregulating α-SMA expression and optimizing the collagen I/III ratio, which is a unique advantage that single/dual-component systems cannot fully replicate. These comparisons demonstrate that increasing compositional complexity from single to multi-component TCM systems progressively enhances antibacterial breadth, anti-inflammatory depth, healing rate, and scar inhibition, while also highlighting the need for more precise assembly strategies to overcome current limitations.

In summary, current research on multi-component and formula-mimicking TCM-based hydrogels appears less refined compared to their single- and dual-component counterparts with well-defined compositions. These complex systems theoretically could replicate TCM’s holistic regulatory advantages. However, current studies still have significant limitations. For instance, the assembly strategies remain oversimplified, predominantly relying on physical mixing or simple encapsulation. This approach lacks precise control over the multi-component interactions, making it challenging to achieve genuine functional assembly. Furthermore, mechanistic understanding is severely underdeveloped. The intricate interaction networks within these complex systems, involving multiple components, multiple targets, and multiple pathways, are difficult to decipher precisely, leaving these systems largely in a “black box” state.

**Table 6:** Comparison of therapeutic efficacy between chitosan-based hydrogels with and without TCM components across hierarchical levels

Active components	Assembly strategy	Antibacterial functional elements	Wound healing performance	Anti-inflammatory mechanism	Scar regulation effect	Ref.
Without TCM components	Ion crosslinking, physical entanglement	Chitosan, AgNPs, TiO_2_, BG	7-day healing rate ~82%, nearly full healing at 14 days	Mild reduction of pro-inflammatory factors (TNF-α, IL-6), no targeted immune regulation	Increased collagen deposition, no targeted scar inhibition	[112–114]
Single-component TCM system	Ion crosslinking, hydrogen bonding, coordination	GA, PUE, tannic acid	Wound area reduced to 2.86% at day 12; 14-day healing rate up to 95%	Direct antioxidant and anti-inflammatory effects of TCM monomers, regulating macrophage polarization, accelerating inflammatory-to-proliferative phase transition	Optimized collagen I/III ratio, intact dermal structure, partial hair follicle regeneration	[44, 116 45, 85, 115,]
Dual-component TCM system	Schiff base bonds, metal-phenol coordination, photo-crosslinking	Ferulic acid, chlorogenic acid, GA	Nearly full wound closure at 13–15 days; 14-day healing rate 92% in diabetic mice	Multi-component synergy significantly downregulates multiple inflammatory factors, promotes M1 to M2 macrophage transition	Enhanced collagen deposition, melanin production inhibition, reduced scar formation	[106–108]
Multi-component/formula-mimetic TCM system	Hydrogen bonding, physical blending, hierarchical co-assembly	HPCE, TEP	Accelerated re-epithelialization, angiogenesis and collagen deposition, full healing 2–3 days earlier than single-component systems	Strong anti-inflammatory effect of TCM extracts, inhibiting multiple inflammatory pathways	Explicit anti-hypertrophic scarring effect (inhibition of α-SMA expression), ordered collagen deposition	[105, 117, 118]

## Conclusion and perspectives

This review systematically collates the global research progress of TCM-based hydrogels for chronic wound healing, and establishes a novel component-number hierarchy as the core design concept. This concept clarifies the progressive patterns in structural design, functional realization, and mechanistic regulation across single-component, dual-component, and multi-component/formula-mimicking TCM-based hydrogels. Single-component systems serve as fundamental models. They reveal the assembly behavior of TCM active ingredients and establish basic structure-activity relationships. Dual-component systems achieve functional integration and intelligent upgrades. This is accomplished through synergistic design and spatiotemporally controlled release strategies. Multi-component and formula-mimicking systems show initial promise. They translate the TCM compatibility principle of EMAC into modern material design. Most existing reviews are limited to descriptive summaries of hydrogel preparation methods and superficial accounts of therapeutic effects. In contrast, our proposed component-number hierarchy framework is the first to systematically decipher the full logical link between the hierarchical assembly of TCM components, hydrogel network structure, physicochemical properties, and in vivo wound healing efficacy. More importantly, this framework builds a direct and operable bridge between classic TCM multi-component compatibility theory and modern hydrogel hierarchical assembly design.

Despite the promising progress in the development of TCM-based hydrogels, three critical unresolved core challenges remain in the field: (1) The assembly strategies for multi-component formula-mimetic systems are still oversimplified, predominantly relying on physical mixing rather than precise functional assembly; (2) The synergistic mechanisms of multi-component TCM systems remain in a “black box” state, with no systematic elucidation of their multi-target regulatory networks; (3) The clinical translation of TCM-based hydrogels faces severe bottlenecks, including undefined regulatory pathways, poor batch-to-batch consistency, and the absence of standardized clinical evaluation systems.

Future development of TCM-based hydrogels should focus on the following directions:

1. Innovation in Precision Assembly Strategies: Future research must move beyond simple physical mixing. It should develop precision assembly techniques based on coordination bonds, covalent bonds, and host-guest interactions. The goal is to achieve precise control over the spatial distribution, release kinetics, and functional synergy of multiple components. This will enable a true transition from “physical mixing” to “functional assembly.”
2. Mechanistic Elucidation and Standardization: Integrating multi-omics analysis, single-cell technologies, and artificial intelligence is crucial. These tools can systematically unravel the multi-target regulatory networks of complex TCM hydrogels during wound healing. Clarifying the intrinsic "structure-property-function" relationships is key. This will help make the mechanisms of TCM formula-based hydrogels interpretable and their quality standardized.
3.  Intelligent Responsiveness and Personalized Therapy: Smart hydrogel systems that respond to dynamic wound microenvironment changes (e.g., pH, ROS, enzymes, temperature) need further development [126]. This will enable precise matching between drug release and the stages of healing. Combining technologies like 3D printing and microfluidics can facilitate the creation of customizable, personalized dressings. These would adapt to different types and stages of chronic wounds
4. Clinical Translation and Industrialization Promotion: Strengthening interdisciplinary collaboration is essential to move TCM-based hydrogels from the lab to the clinic. Key priorities include: (i) establishing head-to-head comparisons between TCM-based and non-TCM-based hydrogels under standardized conditions to substantiate claims of unique advantages; (ii) developing evaluation systems compliant with international regulatory standards (e.g., FDA, NMPA) to define safety, efficacy, and stability; (iii) exploring scalable GMP-compliant manufacturing processes; and (iv) designing well-controlled clinical trials with quantifiable endpoints (e.g., healing time, scar formation rate) to demonstrate translational value.

In conclusion, TCM-based hydrogels act as a bridge connecting traditional TCM wisdom and modern materials science. By deeply integrating the “component-number hierarchy” design concept with the TCM philosophy of EMAC compatibility, these systems are evolving from single-function materials into intelligent, synergistic, and systematic therapeutic platforms. This concept provides a theoretical foundation for the rational design of next-generation chronic wound healing materials, offering new hope for patients worldwide suffering from refractory wounds.

## Data Availability

No datasets were generated or analysed during the current study.

## Abbreviations

AS: Asiaticoside
Ber: Berberine
BSP: Bletilla striata polysaccharide
CA: Chlorogenic acid
CD31: Platelet endothelial cell adhesion molecule-1
CF: Cyclodextrin-based formulation
CMCS: Carboxymethyl chitosan
CS: Chitosan
Cur: Curcumin
EGCG: Epigallocatechin gallate
EMAC: Emperor-minister-assistant-courier
EPL: ε-polylysine
FA: Ferulic acid
GA: Gallic acid
GelMA: Gelatin methacryloyl
GEP: Gastrodia elata polysaccharide
GOx: Glucose oxidase
HACC: Hydroxypropyl trimethyl ammonium chloride chitosan
IPN: Interpenetrating network
Lipo: Liposomes
MN: Microneedle
MOF: Metal-organic framework
MRSA: Methicillin-resistant Staphylococcus aureus
OBSP: Oxidized Bletilla striata polysaccharide
OFu: Oxidized fucoidan
OGEP: Oxidized gastrodia elata polysaccharide
PNIPAm: Poly(N-isopropylacrylamide)
PPD: 20(S)-protopanaxadiol
Pue: Puerarin
PVA: Polyvinyl alcohol
QCS: Quaternized chitosan
QCT/Q: Quercetin
ROS: Reactive oxygen species
SA: Sodium alginate
SIS: Small intestinal submucosa
TA: Tannic acid
TCM: Traditional Chinese medicine
TEP: Tibetan eighteen-flavor dangshen pill
TMP: Tetramethylpyrazine
VEGF: Vascular endothelial growth factor
β-D-PGG: 1,2,3,4,6-penta-o-galloyl-β-D-glucose
γ-PGA: Poly-γ-glutamic acid
